# RNA-Seq Analysis of Oil Palm under Cold Stress Reveals a Different C-Repeat Binding Factor (CBF) Mediated Gene Expression Pattern in *Elaeis guineensis* Compared to Other Species

**DOI:** 10.1371/journal.pone.0114482

**Published:** 2014-12-05

**Authors:** Xintao Lei, Yong Xiao, Wei Xia, Annaliese S. Mason, Yaodong Yang, Zilong Ma, Ming Peng

**Affiliations:** 1 Hainan Key Laboratory of Tropical Oil Crops Biology/Coconut Research Institute, Chinese Academy of Tropical Agricultural Sciences, Wenchang, Hainan, P. R. China; 2 Institute of Tropical Bioscience and Biotechnology, Chinese Academy of Tropical Agricultural Science, Haikou, Hainan, P. R. China; 3 School of Agriculture and Food Sciences and Centre for Integrative Legume Research, the University of Queensland, Brisbane, Australia; Huazhong university of Science and Technology, China

## Abstract

*Elaeis guineensis* as a tropical oil-crop is particularly sensitive to low temperature. Improvement of cold-tolerance may significantly increase the total cultivation area of this tropical oil-crop worldwide. We sequenced cold-treated and control (untreated) samples of *Elaeis guineensis*. *De novo* assembly generated 51,452 unigenes with an average length of 703 bp. Subsequently, these expressed sequences were functionally annotated. In the K category (transcription factors) of COG (Cluster of Orthologous Group) annotation, the largest proportion of genes induced and repressed at least two-fold under cold stress were from the AP2/ERE family, indicating that C-repeat binding factor, (CBFs, members of the AP2/ERE family) may play a central role in cold tolerance in *Elaeis guineensis*. Subsequently, the CBF-mediated signal transduction pathway was reconstructed based on transcriptome data and the gene expression profile involving the pathway was examined using real-time quantitative RT-PCR (qRT-PCR). CBFs reached maximum transcript level both at medium (4 h) and long period time points (7 days), contrary to the expression pattern of CBFs in *Arabidopsis* and rice. Moreover, the promoters of downstream *Cold Responsive* gene (*CORs*) regulated by CBFs were analyzed. Conservation, mutation and absence of the DRE core motif were detected in the promoters of six CORs. These mutations in DRE motifs suggest that CORs may not be induced *via* cold stress in *Elaeis guineensis*.

## Introduction

Oil palm, belonging to the family Arecaceae, is a major tropical oil crop worldwide. In 2012, the total yield of oil palm was about 50 million tons of palm oil from 17 million hectares of plantation (http://faostat3.fao.org/home/E). The genus *Elaeis* contains two species: *E. guineensis* (Africa oil palm) and *E. oleifera* (American oil palm). Compared to other oil crops, *E. guineensis* is the highest oil-yielding crop per unit area in the world. Currently, the oil palm is cultivated mainly in the tropics, including Southeast Asia, Africa, Central America and Brazil. The best mean temperature for oil palm is about 27°C, with a minimal growth temperature of 15°C [1]. Regional trial plantings of oil palm in the south of China and in other countries outside the usual oil palm growing regions are now underway. However, low temperatures in winter adversely affect oil palm growth, fruit development and consequently oil production in these subtropical regions. Low temperatures can also cause cold damage such as yellowing and withering of young leaves and flowers, and slowing of flower bud differentiation. Thus, improvement of cold tolerance of *E. guineensis* would significantly increase the total cultivation area and the total oil production of this oilseed crop.

After plant exposure to cold stress, a series of biochemical and physiological alterations at the molecular and cellular level are induced to aid plant growth and survival under cold stress conditions [2], [3]. These changes include alteration of the composition and structure of the cell plasma, increased concentrations of soluble sugar, sugar alcohols and other low-molecular-weight nitrogenous compounds, decreased free water content and generation of antifreeze proteins [4], [5], [6]. Meanwhile, the expression of particular genes may also be induced in different plant species in response to cold signals [7]. From these induced genes, some gene products directly participate in defense to cold stress, such as *via* biosynthesis of osmotic compounds [8], [9], generation of antioxidants, and increase in membrane fluidity [10], [11]. Other genes may function in regulation of gene expression and signal transduction under cold stress, e.g. transcription factors and proteins involved in RNA processing and nuclear export [12], [13]. Although previous research works has identified physiological alterations in *Elaeis guineensis* under cold stress [14], the molecular mechanism underlying these physiological alterations are still unknown. Deeper understanding of the mechanisms of gene expression regulation and the metabolic profile of *E. guineensis* under cold stress is imperative.

In *Arabidopsis* and rice, the CBF (C-repeat Binding Factor)-mediated signal transduction pathway has been proved to play a central role in cold tolerance. In *Arabidopsis*, the CBF/dehydration-responsive element-binding factor (DREB1) protein family contains three gene members: CBF1/DREB1B, CBF2/DREB1C and CBF3/DREB1A [13], [15]. Transgenic *Arabidopsis* plants constitutively expressing CBF1, CBF2 and CBF3 had increased cold tolerance [16], [17], [18]; while down-regulation of CBF expression by RNA interference and antisense RNA resulted in about a 25% to 50% decrease in freezing tolerance when plants were exposed to low temperatures [19]. The CBF protein can bind to cis-acting element DRE (dehydration-responsive element) or to CRT (C-repeat) so as to activate the expression of cold-responsive genes (*CORs*) which may encode enzymes involved in the biosynthesis of low-molecular-weight nitrogenous compounds, cryoprotectants such as sucrose, raffinose, proline, hydrophilic cryoprotective polypeptides etc [20].

ICE1 (inducer of CBF expression 1), a MYC-like basic helix-loop-helix transcription factor, is a major positive regulator of CBF genes. The constitutive expression of ICE1 will induce CBF3 expression without exposure to low temperature [21]. Miura et al. (2007) provide strong evidence that low temperature-induced sumoylation of ICE1 proteins mediated by SIZ1 proteins are required for activating the expression of CBF genes [22]. Meanwhile, ICE1 proteins can be inactivated due to ubiquitination and protein degradation mediated by HOS1 (High expression of Osmotically responsive genes1), a RING finger E3 ligase. At room temperature, the HOS1 gene is expressed in the cytoplasm. When *Arabidopsis* plants are exposed to cold environments, HOS1 gene expression is found in the nuclei [23], [24]. In *Arabidopsis*, low temperature seems to be able to initiate a cycle of activation and inactivation of ICE1 proteins based the SIZ1-HOS1 system, which contributes to accurately regulate the expression of CBF genes. Moreover, transgenic *Arabidopsis* constitutively expressing MYB15 will lead to a decrease in the expression of CBF genes and reduction of cold tolerance, indicating that MYB15 is a negative regulator of CBF gene expression. Meanwhile, the *ice1* mutation in *Arabidopsis* shows a sustainable increased expression level of MYB15, indicating that ICE1 can negatively regulate the expression of the MYB15 gene [25], [26].

However, in comparison to *Arabidopsis* and cereals crops, *E. guineensis* is a tropical crop which is evolutionarily adapted to tropical environments and which does not undergo cold acclimation processes, making this oil crop sensitive to low temperature exposure. Thus, it is interesting to ascertain the global gene expression change in oil palm when it is exposed to low temperature environments. RNA-seq is a next-generation sequencing biotechnology that can be used for large scale gene discovery. Compared to traditional Sanger sequencing method, RNAseq is simpler and more cost-efficient, as well as highly accurate and sensitive for gene expressions [27]. In this study, RNAseq was used to investigate gene expression changes in *E. guineensis* under cold treatment using Illumina sequencing technology, subsequently deepening our understanding about the molecular mechanisms underlying cold responses in *E. guineensis*.

## Materials and Methods

### Plant material

F1 hybrid seedlings from crosses between oil palm subspecies *dura* (thick-shelled African oil palm) and *pisifera* (thin-shelled African oil palm) were grown in nurseries. The two African oil palm parents o were introduction to China from Malaysia in the 1990s. Twenty one one-year-old F1 hybrid plants germinated in the same week and grown in the same nursery were selected for subsequent cold treatment. Prior to treatment, the hybrid seedlings were placed in a growth chamber at 26°C for one day. Subsequently, spear leaf samples were collected from three individual replicates (as controls) for RNA extraction. The remaining six groups of three seedling replicates were kept at 8°C for 0.5 hours, 1 hour, 4 hours, 8 hours, 1 day and 7 days respectively before sampling. Spear leaves were sampled from control and cold-treated seedlings and immediately frozen in liquid nitrogen.

### RNA extraction

Total RNA was extracted separately from the control and cold treated leaf samples using the MRIP method described by Xiao et al. (2012) [28] and subsequently equally mixed. The MRIP extraction buffer comprised (100 ml): 3.5 g ammonium thiocyanate, 9.44 g guanidine thiocyanate, 3.33 ml 3 mol/L sodium acetate (pH 5.2), and 38 ml phenol, then adjust pH to 5.0. The protocol refers to Xiao et al. (2012) [28]. Total mRNA equally mixed from the control samples was then mixed in equal proportions and prepared for illumina sequencing.

### Illumina sequencing and *de novo* assembly

Purified mRNA isolated from the control sample and from the cold-treatment mixture were separately fragmented with divalent cations under increased temperature. These short fragments were taken as templates to synthesize the first-strand cDNA using hexamer primers and superscript III (Invitrogen, Carlsbad, CA, USA). Second-strand cDNA was then synthesized in a solution containing buffer, dNTP, RNaseH and DNA polymerase I and subsequently purified using a QiaQuick PCR extraction kit (Qiagen). EB buffer was used to resolve these short fragments for end reparation and poly (A) addition. The sequence adaptors were linked to two ends of short cDNA sequences and suitably sized cDNA fragments were selected out for PCR amplification based on the agrose gel electrophoresis results. Finally, the library established was sequenced using an Illumina Hiseq 2000. The paired-end library was developed according to the Paired-End sample Preparation kit protocol (Illumina, USA). The transcriptome short reads were *de novo* assembled using transcriptome *de novo* assembly software following the protocol documented by Grabherr et al (2010) [29], using default Kmer value (K = 25).

### Functional annotation of expressed sequences (Unigenes)

The expression sequences (Unigenes) were blasted against the NR database with an E-value cut-off of 10^−5^ (E-value <0.00001). Subsequently, the expressed sequences were aligned by BLASTX to databases Swiss-Prot, KEGG and COG for putative function annotation. If BLAST results of different databases conflicted, NR then Swiss-prot database results were given precedence. The WEGO software was used to perform GO functional classification of all expressed sequences [30], available at wego.genomics.org.cn/cgi-bin/wego/index.pl. The results of the GO annotation were also used for the KEGG and COG analyses.

### Real-time qPCR assays

Real-time PCR was performed following a standard SYBR Premix Ex Taq kit (TaKaRa) protocol in 96-well optical plates (Axygen) using a final volume of 10 µl. The reactions were incubated in 0.2 ml tubes of a Mastercycler ep realplex4 (Eppendorf) machine as follows: 95°C for 5 s, 55°C for 15 s and 68°C for 20 s. The procedure ended by a melt-curve ramping from 60 to 95°C for 20 minutes to check the PCR specificity. All qPCR reactions were carried out in biological and technical triplicate. The final Ct values were the means of nine values. The comparative expression levels of the selected expressed sequences were normalized to that of EIF, which was previously found to be a stable reference gene under cold stress in oil palm [31].

### Calculating gene expression level

RPKM (Reads Per kb per Million reads) was used to calculate gene expression level using the following formula [32]:
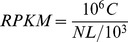



Where C is the number of reads which exclusively aligned to one expressed sequence; N is the total number of reads which were aligned to all expressed sequences and L is the basic number in the CDS of the corresponding expressed sequence.

The statistical significance of the differential expression was determined according to the method documented by Audic and Claverie (1997) [33]. The statistical significance *p(x)* can fit the poisson distribution.
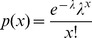



Arabic alphabet x in the formula represents number of reads mapped to unigene. λ refers to the real transcripts of the unigene.

When thousands of hypothesis tests are performed, the *p*-value suitable for a single test is not sufficient to guarantee a low rate of false discovery. Thus, an FDR (False Discovery Rate) control method was applied using multiple hypothesis testing to correct the *p*-value results [34]. Subsequently, the RPKM ratio was used to compute the fold change of gene expression for each pair of samples simultaneously. The differentially expressed genes were selected using a threshold of FDR ≤0.001 and an absolute value of log_2_ratio ≥1 [35].

### Genomic location of putative orthologs involved in the CBF-mediated signal transduction pathway

Putative orthologous genes involved in the CBF-mediated signal transduction pathway were predicted based on annotation results. In order to determine the chromosomal location of these putative orthologs, the sequences of the putative orthologous genes were blasted against the database of oil palm whole-genome shotgun contigs (BioprojectID: 192219: PRJNA192219).

## Results

### RNA-Seq and *de novo* assembly

We generated RNA-Seq data from the control sample and the mixed sample with cold treatment using the Illumina HiSeq2000 genome analyzer. In total, 77 million paired-end reads were produced, with an average read length of 90 bp, (Table 1). All clean reads had been deposited in the National Center for Biotechnology Information (NCBI) (Submission Number: SRR1612397 and BioProject: SRP048913). The Q20 percentages for the cleaned reads were 97.21% and 95.77% for the two samples, indicating that the percentage sequencing error was lower than 1%. Subsequently, *de novo* assembly was performed with Illumina reads using Trinity software.^27^ In total, 51,452 transcripts were obtained, these data had been deposited in TSA (Transcriptome Shotgun Assembly) database of NCBI website (Submission Number: GBSV00000000): 40,725 from the control sample and 49,500 from the mixed sample with cold treatment. The average transcript size was 703 bp and the N50 was 1072 bp (Table 2).

**Table 1:** Summary of RNA-Seq datasets obtained for control and cold-treated *Elaeis guineensis* samples.

	Control sample	Mixed sample with cold treatment	Total
Number of reads (million)	38.68	38.56	77.24
Total bases (raw, Gb)	4.47	4.29	8.76
Total bases (trimmed, Gb)	3.87	3.86	7.73

**Table 2:** Data outcomes for the *Elaeis guineensis* control and cold-treated samples.

	Control sample	Mixed sample with cold treatment	Total
Number of transcripts	40,752	49,500	51,452
Number of transcripts (>1000 bp)	7,909	10,233	10,916
Total base pairs (Mbp)	25.58	32.19	36.15
Average length (bp)	628	650	703
N50 (bp)	871	950	1072

A greater number of transcripts were obtained from the mixed sample with cold treatment (49,500) compared to the control (Table 2). Of these transcripts, 2665 transcripts occurred exclusively in the mixed sample. Longer average transcript lengths (650 bases, N50 = 950) were also obtained from the mixed sample after assembly.

**Figure 1:**
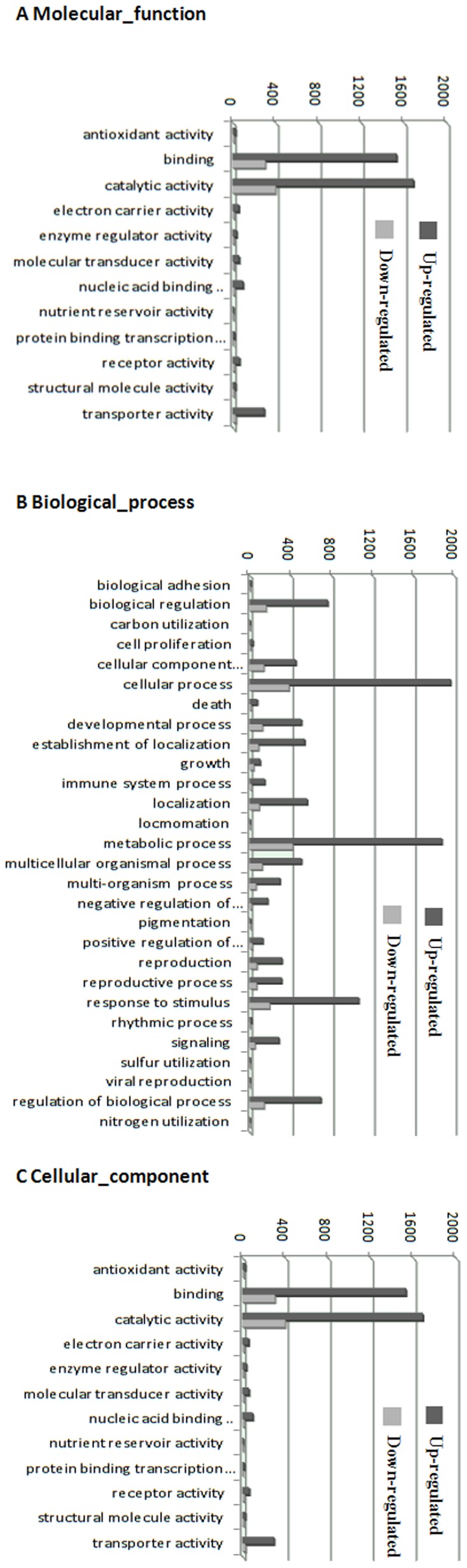
The transcripts classified based on gene ontology (GO) terms for (A) ‘Molecular_Function’, (B) ‘Biological_Process’, and (C) ‘Cellular_Component’ of up-regulated and down-regulated transcripts in response to cold stress.

To explore the biological functions of the cold-responsive genes, a total of 51,452 transcripts were used as queries to perform an alignment with the NCBI non-redundant (NR) protein database, using a cut-off E-value of 10^−5^. Of these transcripts, 37,151 sequences had hits to known protein sequences in the NR database. A total of 27,900 transcripts (54.2%) were assigned at least one GO term. Among these annotated transcripts, 3934 (14.1%) were up-regulated at least two-fold and 742 (2.6%) were down-regulated at least two-fold. These putative cold stress response transcripts could be categorized into 54 GO terms based on the annotation results. Metabolic processes (2299, 8.24%), cellular processes (2348, 8.42%) and catalytic activity (2088, 7.48%) and had the largest number of transcripts putatively produced in response to cold stress based on the biological process and molecular function GO categories, and these were localized in the cell part (2541, 9.11%) cellular component (Figure 1). Based on these results, a large number of transcripts assigned into different GO categories were induced or repressed in response to cold stress, suggestive of comprehensive physiological changes when oil palm suffers cold stress.

A BLAST analysis of the assembled transcripts against the KEGG database matched 22,687 transcripts with corresponding Enzyme Commission (EC) numbers and canonical KEGG reference pathways. 128 biosynthesis pathways were predicted. Differential expression analysis showed that some KEGG pathways were induced in response to cold stress (File S1). Of these cold-response pathways, some had been previously reported to be associated with cold-stress or environmental stress, such as Brassinosteroid biosynthesis, Zeatin biosynthesis, Benzoxazinoid biosynthesis, Flavoneand flavonol biosynthesis and plant-pathogen interactions (for which 33.33%, 31.58%, 26.92%, 21.35% and 18.84% of transcripts respectively were up-regulated at least two-fold). Pathways involving amino acid degradation or metabolism were also induced in response to cold stress, such as Tryptophan metabolism, Glutathione metabolism, Cysteine and methionine metabolism, Lysine degradation, Tyrosine metabolism and Phenylalanine metabolism (for which 25%, 21.99%, 21.43%, 20.78%, 19.23% and 18.59% of transcripts respectively were up-regulated at least two-fold). The involvement of these amino acid degradation and metabolism pathways may indicate that oil palm decreases some polypeptide synthesis to increase tolerance to cold stress. Moreover, mismatch repair, base excision repair and non-homologous end-joining (for which 24.24%, 21.51% and 20.45% of transcripts respectively were up-regulated at least two-fold), were also found to be induced in response to cold stress. These pathways may be involved in decreasing DNA damage when oil palm is suffering from cold stress.

### Transcription factors involved in cold response

**Figure 2:**
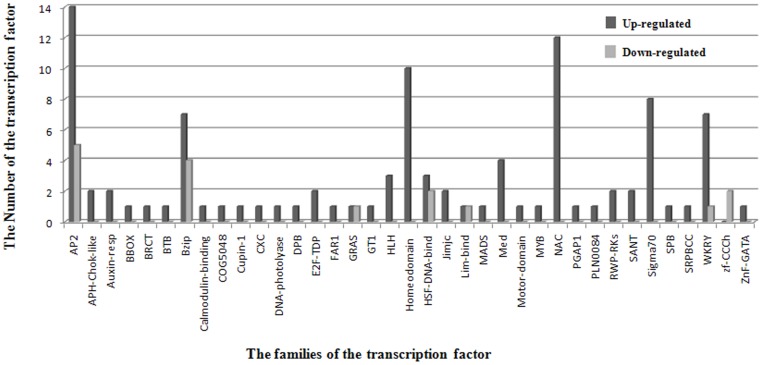
The expression profile of transcription factor families in response to cold stress in oil palm.

A total of 4498 transcripts were identified as transcription factors based on COG annotation (Figure 2). Of these, 293 transcripts were up-regulated at least two-fold, while 97 were down-regulated at least two-fold. Among these cold-responsive transcription factors, the largest gene family was the AP2 family (the family containing the CBF gene, crucial for cold-resistance), within which 15 transcripts were up-regulated at least two-fold and 4 were down-regulated at least two-fold. The second largest gene family was the NAC family, whose transcription factors are associated with cold-resistance in plants and of which 12 transcripts were up-regulated at least two-fold. The bZIP gene family is also associated with cold tolerance, and 7 transcripts from this family were up-regulated at least two-fold, and 4 were down-regulated at least two-fold.

### The CBF-mediated signal transduction pathway in oil palm

Transcription factors with the AP2 domain comprised the largest proportion of transcription factors detected to be induced under oil palm cold stress conditions, of which *CBF/DREB1* (c-repeat-binding factor) is an important member. Hence, the CBF-mediated pathway may play a central role when oil palm is exposed to cold stress. Transcriptome data was used to reconstruct this signal transduction pathway in *Elaeis guineensis via* matches to known genes in this pathway (Table 3).

**Table 3:** Expressed sequences matched to known gene sequences in the CBF-mediated signal transduction pathway.

Expressed sequences	Matched to species	putative ortholog	Identity	E-value
Unigene615	*Vitis vinifera*	ICE1	63%	3.00E-178
Unigene5046	*Vitis vinifera*	ICE1	56%	2.00E-152
Unigene21287	*Vitis vinifera*	ICE1	97%	2.00E-09
Unigene2502	*Vitis vinifera*	SIZ1	57%	7.00E-55
CL1094.Contig1	*Brachypodium distachyon*	SIZ1	60%	0
CL1094.Contig3	*Brachypodium distachyon*	SIZ1	53%	7.00E-31
Unigene6283	*Zea mays*	MYB15	42%	1.00E-63
Unigene8210	*Vitis vinifera*	HOS1	71%	1.00E-84
CL4552.Contig1	*Sabal minor*	CBF	86%	6.00E-101
CL4552.Contig2	*Ravenea rivularis*	CBF	93%	8.00E-33
CL6255.Contig2	*Glycine max*	CBF	61%	2.00E-42
CL4.Contig1	*Zea mays*	CBF	73%	1.00E-59
CL83.Contig2	*Dypsis lutescens*	CBF	60%	9.00E-62
CL83.Contig3	*Ravenea rivularis*	CBF	55%	6.00E-58
Unigene26961	*Zea mays*	CBF	76%	3.00E-35
CL2890.Contig1	*Vitis vinifera*	CBF	53%	1.00E-58

**Figure 3:**
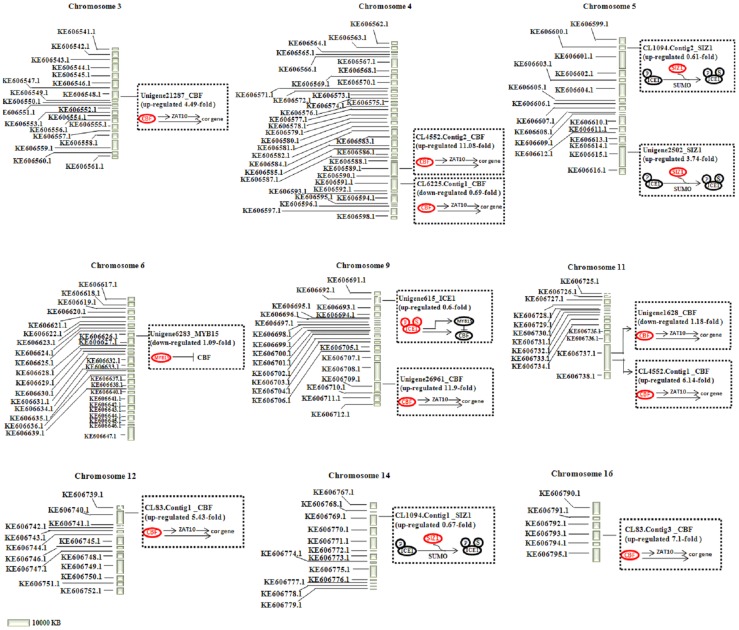
Chromosome positions of the expressed sequences involved in the CBF-mediated signal transduction pathway. The left number “KE……” represents the scaffold ID number in the *Elaeis guineensis* genome in the NCBI database. Chromosomes are comprised of *Elaeis guineensis* scaffolds and the spaces between the scaffolds represent gaps between the scaffolds. A length of 1 centimeter represents 10000 kb.

Based on transcriptome data in response to cold stress, three expressed gene sequences (Unigene615, Unigene5046 and Unigene21287) showed high similarity to known ICE1 gene sequences which may participate in activating CBF gene expression when oil palm is exposed to low temperatures. Using *in-silico* mapping, Unigene615 and Unigene21287 were located on to chromosome 9 and chromosome 3 of oil palm respectively (Figure 3). Moreover, Unigene2502, CL1094.Contig1, and CL1094.Contig3 also showed high similarity to known SIZ1 gene sequences: SIZ1 can facilitate conjugation of SUMO to ICE1 proteins to activate the CBF gene expression. These three expressed sequence were located on chromosome 5 (Unigene2502), chromosome 14 (CL1094.Contig1), and chromosome 5 (CL1094.Contig3). Meanwhile, the conjunction of SUMO and ICE1 protein can also repress the expression of the MYB15 gene (a repressor of CBF gene expression). The expressed sequence Unigene6283 showed high similarity to the known MYB15 gene sequence, and was mapped to chromosome 6. ICE1 proteins are ultimately degraded by HOS1-mediated ubiquitination. Based on the transcriptome data, one expressed sequence (Unigene8210) showed high similarity to known HOS1 gene sequences, but could not be mapped to a chromosomal location in oil palm.

A total of 8 expressed sequences (CL2890.Contig1, CL4552.Contig1, CL4552.Contig2, CL6255.Contig2, CL4.Contig1, CL83.Contig2, CL83.Contig3, and Unigene26961) showed high identity with known *cbf* gene sequences. 5 of these expressed genes were up-regulated at least two-fold in the mixed sample with cold treatment comparative to the control sample. The 8 expressed sequences were located on chromosome 11 (CL4552.Contig1), chromosome 4 (CL6225.Contig1 and CL4552.Contig2), chromosome 2 (CL4.Contig1), Chromosome 12 (CL83.Contig1), Chromosome 16 (CL83.Contig3), and chromosome 9 (Unigene26961). CBF proteins can activate the expression of ZAT10 or activate the expression of the downstream genes (cold-regulated genes). Based on transcriptome data, two expressed sequences (Unigene 713 and CL1971.Contig2) were highly similar to the known ZAT10 gene sequences, and could be mapped on to chromosome 5 and chromosome 15 of oil palm respectively. However, no expressed sequences were identified for the characterized ZAT12, which participates in hindering the expression of *cbf* genes. Meanwhile, a los2 ortholog, which is a repressor of ZAT10, was also not detected based on the transcriptome data in response to cold stress.

**Figure 4:**
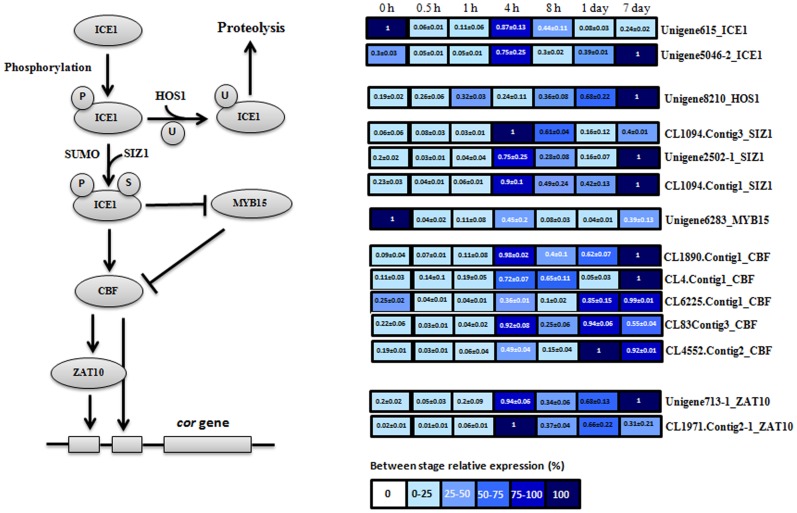
The expression profile of 15 unigenes putatively involved in the CBF-mediated signal transduction pathway at 0 h, 0.5 h, 1 h, 4 h, 8 h, 1 day and 7 days after cold treatment. Gene expression levels at 0 h, 0.5 h, 1 h, 4 h, 8 h, 1 day and 7 days after cold treatment are indicated with colored bars. The number within the color bar indicates the average value of the relative expression level and the standard deviation (average value ± standard deviation) at that time point.

In order to examine gene expression patterns involving the CBF-mediated pathway, quantitative real-time PCR (qRT-PCR) was used to detect expression changes in 14 unigenes (5 *cbf* orthologs, 2 *ice1* orthologs, 3 *siz1* orthologs, 2 *zat10* orthologs, 1 hos1 ortholog, and 1 *myb15* ortholog) at 0 h, 0.5 h, 1 h, 4 h, 8 h, 1 day and 7 days after cold treatment (Figure 4). At 0 h after cold treatment, all unigenes showed low expression levels except for Unigene615_ICE1 and Unigene6283_MYB15. It is possible that a large amount of MYB15 protein at this stage could hinder the expression of the *cbf* gene under warm conditions (27°C). Moreover, although Unigene615_ICE1 had a high expression level at this stage, the ICE1 protein cannot be sumoylated to avoid activation of the *cbf* gene due to low *SIZ1* gene expression levels at this stage. At the following two time points (0.5 h and 1 h after cold treatment), all unigenes showed low expression levels. However, the expression levels of *ICE1* (Unigene615_ICE1 (0.87±0.13) and Unigene5046-2_ ICE1 (0.75±0.25)) and *SIZ1* (CL1094.Contig3_SIZ1 (1), Unigene2502-1_SIZ1 (0.75±0.25), and CL1094.Contig1_SIZ1 (0.9±0.1)) genes were sharply increased at 4 h after cold treatment. Obviously, the expression of *ICE1* and *SIZ1* can lead to the sumoylation of the ICE1 protein so as to activate *CBF* gene expression at this stage. The expression of CL1890.Contig1_CBF (0.98±0.02), CL4.Contig1_CBF (0.72±0.07) and CL83.Contig3_CBF (0.92±0.04) was intensively induced at this stage according to quantitative real-time PCR results. Meanwhile, Unigene6283_MYB15, a repressor of *CBF* gene expression, also showed increasing expression level at this stage, and may have reached highest expression level between 4 h and 8 h. The accumulation of CBF protein can activate the expression of the downstream *ZAT10* genes. In fact, two *ZAT10* genes (Unigene713-1_ZAT10 (0.94±0.06) and CL1971.Contig2-1_ZAT10 (1)) were intensively induced at this stage based on the quantitative real-time PCR results. Subsequently, the expression of almost all genes decreased at the following time point (8 h after cold treatment). However, at the last two time points (1 day and 7 days after cold treatment), the expression of *ICE1* genes and *SIZ1* genes had gradually increased, which resulted in high expression levels of *CBF* genes at 7 days after cold treatment. At this stage, high expression levels of the *HOS1* gene ortholog (Unigene8210 (1)) were also detected, which would play a positive role in hindering the expression of the *CBF* gene by degrading the ICE1 protein. In brief, different to in *Arabidopsis* and rice, the expression of *CBF*s in *Elaeis guineensis* reaches a maximum level both at medium (4 h) and long (7 day) periods after cold treatment.

### AP2 domains of the CBFs in *Elaeis guineensis*

**Figure 5:**
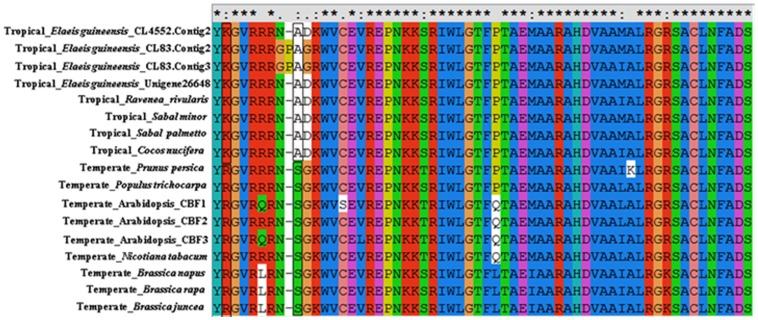
Alignment of AP2/ERE domain amino acid sequences from *Elaeis guineensis* and other species from different climatic zones.

In plants, the AP2/ERE domain of the CBF protein is mainly responsible for binding the cis-element of the COR gene. In order to ascertain if the AP2/ERE domain is conserved in this tropical oil-crop, AP2/ERE domain amino acid sequences from *Elaeis guineensis* were aligned with those in other species from different climatic zones (Figure 5). High similarity was found between AP2/ERE domain amino acid sequences from *Eleais guineensis* and *Arabidopsis*: sequence identity varied from 83% to 86%. Meanwhile, 45 (75%) of the 60 amino acids were identical between AP2/ERE domains from palm species and temperate-climate species. Interestingly, position 2 and position 9 of the AP2 domain were K (Lys) and A (Ala) respectively for palm species, while in the other, temperate climate species these positions were R (Arg) and G (Gly). Other than that, the sequence of the AP2 domain was highly conserved between *Elaeis guineensis* and the temperate climate species, implying functional conservation of the AP2 domain in *Elaeis guineensis*.

### Analysis of CORs and their promoters in *Elaeis guineensis*

CBFs can specifically bind to cis-acting elements of the DRE/C-repeat domain in the promoter region of COR genes. In this study, six COR genes were identified based on transcriptome annotation results for *Elaeis guineensis*, including CL4270.Contig1, CL4270.Contig2, CL4270.Contig3, Unigene10238, Unigene16556, and CL1557.Contig1. The expression levels of the six CORs were all up-regulated under cold stress. Meanwhile, the properties of the proteins encoded by these COR genes were also analyzed. The hydrophilins of these proteins varied from 0.03 to 0.821 (Table 4).

**Table 4:** Mean hydrophilicity, expression change and cis-elements of six COR genes in *Elaeis guineensis*.

Unigene	Hydropathicity	DRE motif	Distance between DRE and strat code	Fold change	Matched species
CL4270.Contig1	0.75	no DRE motif		0.045	*Ricinus communis*
CL4270.Contig2	0.821	G/ACCGAC	218 bp	0.045	*Zea mays*
CL4270.Contig3	0.749	CCCGAC	370 bp	0.045	*Ricinus communis*
Unigene10238	0.641	CCCGAC	404 bp	0.1904	*Oryza sativa*
Unigene16556	0.437	G/ACCGAC	1256 bp	1.3427	*Triticum aestivum*
CL1557.Contig1	0.03	no DRE motif		0.37	*Jatropha curcas*

In *Arabidopsis*, the DRE core motif is A/GCCGAC, which is responsible for the binding of CBF3/DREB1A. In order to ascertain if there was also a DRE core motif in the promoter of the six CORs in *Elaeis guineensis*, the sequences of the six CORs were aligned with genome contigs of *Elaeis guineensis* and 2000 bp upstream of the start codon of the six CORs were subsequently analyzed (Table 4). The DRE core motif (G/ACCGAC) could be found in promoters of CL4270.Contig2 and Unigene16556 218 bp and 1256 bp away from the start codons respectively. However, compared to the DRE core motif (G/ACCGAC) in *Arabidopsis*, single nucleotide variations (CCCGAC) were detected in the promoters of CL4270.Contig3 and Unigene10238. DRE core motifs were also completely absent in the promoters of CL4270.Contig1 and CL1557.Contig1. Because of these mutations in the DRE motif, some CORs may not be inducibly expressed under cold stress in *Elaeis guineensis*.

## Discussion

*Elaeis guineensis* is an important tropical oil crop. As the highest oil-yielding plant in the world, the oil palm is also known as the ‘oil king’. However, the plantation areas of oil palm are limited to tropical countries, in particular Indonesia and Malaysia. In order to increase the total cultivation area and subsequent oil production from *Elaeis guineensis*, some effort has been made to introduce oil palm into subtropical regions, such as the Yunnan and Hainan provinces of China. However, low temperatures in winter in these subtropical regions leads to the significant reduction of flesh fruit product in *Elaeis guineensis*. Here, we performed the analysis of differential gene expression based on transcriptome sequencing of two RNA samples, a control sample and a mixed sample with cold treatment. A total of 51,452 transcripts were obtained, with an average length of 703 bp per transcript. Subsequently, the expressed sequences were aligned with NR, Swiss-Prot, KEGG and COG databases for putative functional annotation. Based on KEGG annotation, a large proportion of genes involved in the biosynthesis of cold-tolerance molecules, amino acid degradation and metabolism and DNA repair were induced in response to cold treatment. Meanwhile, based on the COG annotation, a large number of AP2 domain-containing transcription factors (such as CBF) were induced or repressed at least two-fold. Based on the RNA-seq data and qRT-PCR analysis, we reconstructed the CBF-mediated signal transduction pathway and elucidate gene expression patterns involving this pathway in oil palm.

Recently in plants, there has been an increasing interest in the application of transcriptome sequencing to elucidate the molecular mechanisms associated with traits of interest [7], [36], [37]. Similarly, in *Elaeis guineensis*, some effort has also been made to gain insight into the molecular mechanisms underlying tissue culture response and fatty acid biosynthesis using transcriptome sequencing technology [38], [39]. For example, Low et al. (2008) obtained 9,584 putative unigenes based on random sequencing of clones from a total of 12 standard cDNA libraries [40], representing three main developmental stages in oil palm tissue culture. Tranbarger et al. (2011) obtained 29,304 contigs based on 454 pyrosequencing from the four different stages of mescocarp development [41]. In the present study, Illumina sequencing technology was applied to elucidate the cold-responsive molecular mechanisms of *Elaeis guineensis*. A total of 51,452 transcripts were obtained, similar to the number of transcripts obtained by Illumina sequencing in other species [42]. However, compared to previous sequencing of cDNA libraries and 454 pyrosequencing in *Elaeis guineensis* [40], Illumina sequencing both produced a larger quantity of expressed sequence data and was more cost-effective for transcriptome analysis in *Elaeis guineensis*.

Cold stress, which can be classed as either cold chilling (<20°C) or freezing (<0°C), has a negative effect on the growth and development of plants and can lead to dramatic changes in metabolic reactions, influencing water and nutrient uptake, membrane fluidity and protein and nucleic acid conformation.^13^ Some molecules can be biosynthesized to help plant species to survive in cold stress. In the present study, the expression of some genes involved in the biosynthesis of plant hormones was induced under cold stress, including Brassinosteroid and Zentin genes. Kagale et al. (2006) reported that Brassinosteroids can confer cold tolerance in *Arabidopsis thaliana* and *Brassica napus* and increase the stability of the cell membrane [43]. Zeatin has also been shown to be related to biotic and abotic stress in plant systems [44]. Moreover, the expression of some genes involved in the biosynthesis of some secondary metabolites related to biotic and abotic stress were also induced in *Elaeis guineesis*, including Benzoxazinoids and Flavonoids [45], [46]. As well, low temperatures can lead to DNA damage and protein degradation, eliciting pathways that trigger repair functions [47]. Based on KEGG annotation of *Elaeis guineesis*, some genes involved in DNA repair were inducibly expressed by the cold treatment. Meanwhile, biosynthesis of some proteins may stop under cold stress, as some pathways involved in the degradation and metabolism of amino acids were also induced based on KEGG annotation of the cold-responsive transcriptome in *Elaeis guineesis*. Thus, although *Elaeis guineensis* is a tropical oil-crop and sensitive to low temperatures, there seem to be some similarities in molecular mechanisms of cold tolerance between this tropical oil-crop and other temperate-climate plants.

CBF/DREB1 (C-repeat binding factors, also known as dehydration-responsive element-binding proteins) confer cold tolerance and tolerance of drought stress in various plant species [48], [49], and can be induced by cold stress, operating by binding to cis-elements of COR genes. CBF orthologs have previously been isolated from both cold-tolerant and cold-sensitive crops [14]. Overexpressing *Arabidopsis* CBFs in other plant species can enhance chilling/freezing tolerance [16], [17], [18]. Conversely, the ectopic expression of CBF genes from different species can also increase the chilling/freezing tolerance of transgenic *Arabidopsis* [12], [50]. In *Arabidopsis*, the expression of CBF genes reached a maximum level about 2 to 3 hours after cold treatment (4°C), subsequently followed by a decrease to levels that are only a few fold higher than those detected in warm-grown *Arabidopsis*. In the present study, CBF orthologs were found to have high transcript level about 4 hours after cold treatment (8°C), subsequently followed by a decrease of CBF gene transcript levels, which is similar to other species. However, the expression of the CBF orthologs again reached a high transcript level 7 days after cold treatment (8°C). Strong evidence suggests that low temperature-induced sumoylation of ICE1 proteins mediated by SIZ1 proteins is required for activating the expression of CBF genes [22]. In *Elaeis guineesis*, the ICE1 and SIZ1 orthologs also showed high transcript levels at 4 h and 7 days after cold treatment (8°C), linked to high transcript levels of CBF genes at the two time points. In *Arabidopsis*, CBF genes can be negatively regulated by MYB15, an R2R3-MYB family protein. *myb15* mutations show increasing expression levels of CBFs under cold acclimation and enhanced freezing tolerance. In transgenic *Arabidopsis*, overexpressing MYB15 depressed the expression level of CBFs and led to a reduction in freezing tolerance [25], [26]. In *Elaeis guineesis*, MYB15 seems to reach its highest transcript level between 4 h and 8 h after cold treatment, potentially down-regulating CBF expression in that time period. Moreover, in *Arabidopsis*, the SIZ1-HOS1 system also contributes to accurate tuning of CBF expression. Although increased transcript levels were not detected between 4 h and 8 h after cold treatment, the highest HOS1 transcript level was observed 7 days after cold treatment, which would negatively regulate the expression of CBFs at that time point.

The CBF protein can bind DRE core motifs within COR gene promoters in *Arabidopsis* and subsequently initialize defense mechanisms in response to low temperatures [20]. DRE core motifs were first isolated from the promoter of COR15A in *Arabidopsis* [51]. Generally, COR proteins are characterized as strongly hydrophilic and with good thermal stability. In the present study, six CORs were identified and their hydrophilic properties were analyzed. Four of the six CORs showed high hydrophilic properties, with average hydrophilicity ranging from 0.641 to 0.821. Thomashow et al. (2001) identified four CORs, including COR616, COR15a, COR47, and COR78, all containing the DRE core motif ((G/ACCGAC)) in the promoter region [52]. In the present study, six COR promoters were analyzed. Only two of the six contained DRE core motifs located onto their promoters. Single nucleotide mutations and loss of the DRE core motif was in the promoters of other four CORs, which may be due to the fact that *Elaeis guineensis* is grown in tropical environments and therefore is not under natural selection pressure for tolerance of low temperature stress. The mutations observed in the promoters of these CORs may mean that CORs cannot be induced by low temperatures in *Elaeis guineensis*, thus contributing to low-temperature sensitivity in this tropical oil-crop.

## Supporting Information

File S1 **The number of cold-induced and cold-repressed transcripts in biosynthesis pathways.**
(XLSX)
